# Miscellaneous

**Published:** 1886-12

**Authors:** 


### MISCELLANEOUS.

### INDIANS IN MR. BEECHER'S CHURCH.

WARMLY COMMENDED BY THE PASTOR—THEIR MEETING IN THE BROADWAY TABERNACLE.

Half a dozen Indians occupied a front pew in Plymouth Church yesterday morning, and listened intently to Mr. Beecher as he preached upon the hatred and intolerance which characterized much religion. He said that men hated most fervently when they believed they were obeying the voice of conscience as the voice of God, when half of the time it was the voice of the devil. The Jews and the Samaritans hated each other heartily, for they had different views about religion. In fact, added Mr. Beecher, "They both worshipped God like the devil." In regard to himself, Mr. Beecher said that if some persons were orthodox, he was not orthodox. But the only orthodoxy he held was that of love to God and men. He had come through years and much struggle, for he loved the faith of his Father, to a wide sympathy and love for all men and a belief that the Divine love was above all creeds and dogmas.—*N. Y. Tribune*, Nov. 22.

### LADY DOCTORS IN THE FIFTEENTH CENTURY.

Dr. Horowitz, of Frankfort-on-the-Main, has (*The Jewish Chronicle*, May 14, 1884), published a work entitled "Judische Aerzte in Frankfurt," in which the learned author mentions the interesting fact that, as long as four hundred and fifty years ago, Jewesses practiced medicine in that city; they especially devoted themselves to ophthalmia. The female oculist, Dr. Zerlin, whom we meet with in the volume, as having practiced in the year 1428, ventured to reside outside the Judengasse, and believed that she could claim exemption from the payment of taxes on account of her talent, and the general esteem in which she was held. The municipal council rejected her application, and, in 1489, they ordered that Jewish lady doctors should either quit the city or pay taxes like other Jews. A Jewish doctress was, however, more fortunate in the year 1494; she was relieved from the payment of "sleeping-money," a tax imposed on foreign Jews for every day that they remained in Frankfort. With this exemption was coupled an official recognition of her profession, which was of the utmost advantage to the lady.—*Medical Record.*

CAN'T BE TRUSTED.—"I tell you, sir, no woman can be fully trusted," exclaimed a cynical man to a friend. "Why, just look at poor Sniffson. Didn't he love that wife of his? Didn't he think that nothing was too good for her? And how she has requited him!" "How?" asked the other. "Gone and had twins—these hard times."

"SEE here," said an angry citizen to a quack doctor, "that 'wonderful discovery' of yours for preserving the scalp is a fraud."

"Why so?"

"Look at that," he went on, removing his hat, "since using it I have lost all my hair."

"Oh, it doesn't pretend to preserve the hair," replied the doctor, "only the scalp. You've got your scalp left. You mustn't expect too much of medical science."

COL. INGERSOLL gives the following beautiful tribute on the fidelity of woman :—"I tell you women are more prudent than men. As a rule, women are more truthful than men, and ten times as faithful. I never saw a man pursue his wife into the very ditch and dust of degradation and take her in his arms. I never saw a man stand at the shore where woman had been morally wrecked, waiting for the waves to bring back even her corpse to his arms, but I have seen women do it. I have seen women with their white arms lift man from the mire of degradation and hold him to her bosom as though he was an angel."

---

UNAPPRECIATED GOODNESS.—Mr. C—— was pastor of a Baptist church in a certain town in one of the Western States. He had been on very bad terms with his flock for some time. They abused him whenever they could find occasion, and he reciprocated with equal readiness. Before his contract with the parish expired, he received the appointment of Chaplain at the State Prison. Elated at this lucky opportunity of getting rid of him, the congregation came in full numbers to hear his farewell sermon, perhaps less to compliment than to annoy him with their presence. Great was their astonishment, and still greater their anger, when the reverend gentleman chose for his text the following words: "I go to prepare a place for you * * that when I am there ye may be also."—*Carrier Dove.*

---

HOW TO FIGHT A HORNET.—"Do you know," said a gentleman who is authority, in a lecture at the Philadelphia Zoo, "that a hornet is the most singular animal, and the most unpleasant one to fight with, the people ever knew? A person who is stung by a hornet feels ready for a howling war dance, and it all comes from not understanding one disagreeable characteristic of them. If a man was sharp, a hornet could never sting him. The minute a man strikes or throws a stick at him he instantly responds by making for him in a line as straight as the range of a theodolite. And he always gets there. But if the man saw him coming, and only had sense enough to dodge him, the hornet wouldn't get there. And what is more peculiar, the hornet would go straight back to the identical point he started from and make another sally for his victim. But just as often as the man kept dodging he would never get stung. It only requires a man with nerve enough to dodge quicker than the hornet can fly to make it a very interesting combat."—*Ex.*

---

### TWO RIVAL M. D'S.

Two wise M. D's. of rival schools
Oft held dispute of drugs and doles,
Till one of something like a gout
Fell sick, and stopped their falling out.

Then came the other to his side,
His tongue surveyed, his pulses tried ;
Some physic tasted with grimace,
Enquired what Doctor had the case,
And questioned in familiar sort
If he were dosing as he ought.

The sick man answered from his bed,
"I'm doctoring myself," he said.

"O! reckless man," came swift reply
"Change Doctors! else prepare to die."

